# Implementing a 3D printing service in a biomedical library

**DOI:** 10.5195/jmla.2017.107

**Published:** 2017-01

**Authors:** Verma Walker

## Abstract

Three-dimensional (3D) printing is opening new opportunities in biomedicine by enabling creative problem solving, faster prototyping of ideas, advances in tissue engineering, and customized patient solutions. The National Institutes of Health (NIH) Library purchased a Makerbot Replicator 2 3D printer to give scientists a chance to try out this technology. To launch the service, the library offered training, conducted a survey on service model preferences, and tracked usage and class attendance. 3D printing was very popular, with new lab equipment prototypes being the most common model type. Most survey respondents indicated they would use the service again and be willing to pay for models. There was high interest in training for 3D modeling, which has a steep learning curve. 3D printers also require significant care and repairs. NIH scientists are using 3D printing to improve their research, and it is opening new avenues for problem solving in labs. Several scientists found the 3D printer so helpful they bought one for their labs. Having a printer in a central and open location like a library can help scientists, doctors, and students learn how to use this technology in their work.

## BACKGROUND

Three-dimensional (3D) printing applications in biomedical fields are only limited by the imagination and a few technical issues. Although transplantable 3D organs are years away, progress is being made, as demonstrated in 2016 when scientists announced they used a 3D printer to create an ovarian implant for a mouse that produced healthy offspring [1]. Also, small samples of liver, skin, and neural tissues have been 3D printed for toxicology testing and research purposes [2–6]. Developments have not been limited to the lab, as patients, especially those without optimal treatment options, are also seeing the benefits of 3D printing. For instance, patient-specific hip replacements and spinal implants are on the way [7–9]. Another area of strong development is surgical guides that can be designed from medical imaging for a wide range of applications. These guides improve surgical planning, especially for complicated cases, and can shorten surgical time [10–12]

Hoy states that it is possible for doctors and medical students to learn the basic skills for 3D printing and modeling in a library with a consumer-grade printer, which is the type commonly purchased by libraries [13]. These skills can then be used for more sophisticated applications, such as printing a custom hip replacement on a titanium printer or bioprinting. During the recent Science in 3D: 2015 Bioinformatics Festival, sponsored by the National Institute of Allergy and Infectious Diseases, Office of Cyber Infrastructure and Computational Biology, a panelist pointed out that 3D-printing skills are not being taught in medical or dental schools where they are needed to feed future growth in this field [14]. In fact, it is interesting to note how short the leap is from consumer-level plastic printing to bioprinting. A Makerbot Replicator 2x designed to print plastic was modified into a bioprinter in January 2015 to print tracheal cartilage and again in October 2015 to print soft tissues [15, 16].

Four previously published articles provide advice for planning and implementing a 3D printing service in an academic library that can be applied to a medical library setting [17–20]. Library staff at three of these institutions ran the printer, while staff at the University of Alabama used a self-service model after training [17–20]. Although only one article mentions significant maintenance issues, all four agree that there is a significant learning curve for modeling software that should be addressed with user training [17]. The authors of *Academic Library Use of 3D Printing* surveyed twenty-five colleges about their experiences and provided valuable information for librarians considering implementing a printing service [21]. The surveyed institutions answered common questions that librarians starting a 3D printing service would ask, such as: How much do consumables cost in a year? How many staff-hours per month of staff time are needed?

Health sciences librarians are beginning to take notice of 3D printing with discussion at conferences [22–24]. In 2014, the Association of Academic Health Sciences Libraries conducted the 3D Printing Labs in Health Sciences Libraries Survey [24]. When asked, “Do you have a 3D printing lab or offer 3D printing services in your library?,” 5% of respondents said yes, and 40% said they were working to set up such a service.

## STUDY PURPOSE

The National Institutes of Health (NIH) Library ran a 3D-printing pilot to provide NIH staff with an opportunity to try out 3D-printing technology and to gauge their interest in a permanent 3D-printing service. An additional goal of the pilot was to build partnerships among NIH scientists, fellows, students, engineers, and administrators who were interested in 3D printing, so that the library could serve as an information hub connecting individuals who have 3D printing needs to experts in 3D modeling and printing.

## CASE PRESENTATION

After consulting with librarians at other institutions and reading product reviews of consumer-grade 3D printers, the NIH Library purchased a single-nozzle Makerbot Replicator 2 printer that uses a type of plastic known as polylactic acid (PLA). The print size limitations for this model are 11×6×6 inches. Makerbot provides a free proprietary 3D model slicing program, Makerbot Desktop, on its website.

Beginning in June 2014, use of the 3D printer was freely offered to NIH scientists, fellows, and students, and library policy restricted projects to NIH-related work. Due to long print times (average eight to twelve hours), the printer was available on a first-come, first-served basis. To decrease the upheaval to library staff and to provide users with a maximum learning opportunity, the library implemented a self-service model. Users were required to attend a biweekly librarian-led 3D printing orientation on how to prepare models, safely operate the printer, and start print jobs.

A librarian provided continuing assistance by appointment or walk-in, including troubleshooting failed prints and advising on model design and issues. A website was created to help users find information on modeling software and 3D printing at the library, and a Twitter account (@NIHL3D) was established for those who wished to follow new developments in biomedical 3D printing. Users of the 3D printer were asked to fill out a survey (on paper or via SurveyMonkey) including questions about future service model preferences. In addition, library staff reached out to other people at NIH who were interested in or had experience with 3D printers and set up an email discussion list to share expertise and hardware.

Knowledge about the availability of the 3D printer mainly spread by word of mouth. The first user was a research fellow who needed to print a prototype of a drosophila container for drug testing in her lab. Soon, the library was nearly overwhelmed by people interested in the new technology. When the printer broke down and was sent to the manufacturer for repairs, one of the library’s network partners lent his personal Makerbot Replicator 2x to the library in June 2014 so that the pilot could continue. Both printers remain in the library today.

At the end of the pilot period in December 2014, 213 people from 21 different institutes had attended an orientation, with the highest attendance from members of the National Cancer Institute, National Institute of Diabetes and Digestive and Kidney Diseases, and National Institute of Neurological Disorders and Stroke. Based on personal records kept by the librarian, approximately 173 successful print jobs were completed, although many failed the first time. As the printers were available on nights and weekends, the actual number of prints was probably higher. During the pilot period, replacement parts and repairs included:

- nozzle clearing (regularly)
- Z axis chip
- thermocouple (3 times)
- XYZ cable (2 times)
- misaligned gantry
- thermal insulation (3 times)
- loose belt
- motherboard (2 times)
- X axis motor
- 3D printing and replacement of the broken air duct (2 times)

Based on the high usage of the 3D printers during the pilot period, the service became permanent in 2015, and changes were made to the service model to improve its sustainability. A small group of librarians and reference assistants learned to operate and repair the printers so that a greater number of staff could assist with minor problems; orientations were reduced to monthly; hours of printer operation were recorded on a monthly basis; and a voluntary sign-in log was set up to track the number of separate printing jobs. In 2015, 182 people attended orientations, and at least 196 models were produced. Replacement parts and repairs needed for 2015 were:

- nozzle clearing (regularly)
- thermocouple (2 times)
- thermal insulation (impossible to keep on, now runs fine without it)
- motherboard (1 times)
- LCD display (2 times)
- extruder (2 times)
- 3D printing and replacement of the broken air duct (many times, now runs fine without it)

**Figure 1:**
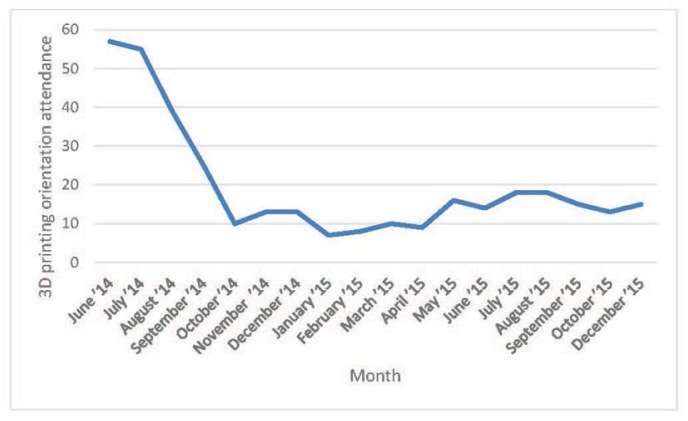
Attendance at 3D printing orientations

Although attendance at the 3D-printing orientations was initially very high, it dropped off after August 2014 (Figure 1), possibly because (1) early adopters were more likely to attend the initial orientations; (2) there was high interest among summer students who left at the end of August 2014; and (3) the number of orientations was reduced at the end of the summer of 2014. Although the library did not actively market the 3D-printing service in 2015, attendance held steady with an average of thirteen people per orientation.

**Figure 2:**
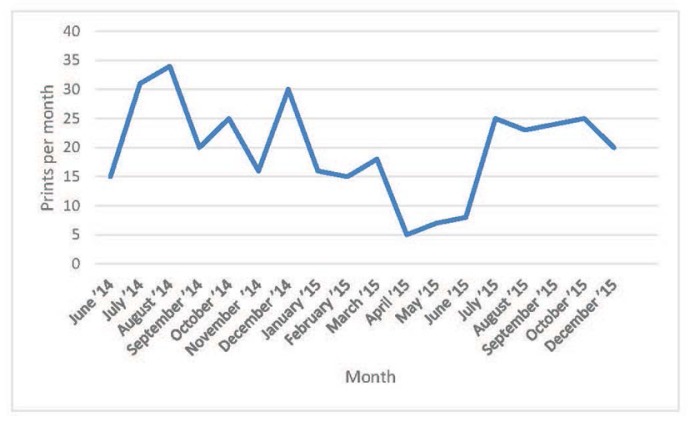
Number of 3D print jobs per month

The number of print jobs per month was erratic (Figure 2), with a substantial decline in April and May 2015 due to maintenance issues with both printers. The most frequently printed 3D models at NIH were prototypes of custom lab equipment. Users also printed models of viruses and proteins from the 3D Print Exchange or the Protein Data Bank, as well as models of brains and lungs with tumors from magnetic resonance imaging and computed tomography scans. To further understand how scientists, fellows, and students used the 3D printers and their opinions on service models, staff surveyed 82 unique users and obtained 32 responses (39% response rate), providing responses to the question of how the printed models would help with their work or research at NIH, such as:

I have been building custom pieces needed for electrophysiological recordings. These pieces included a holder for electronic valves, holders for switches and other electronic equipment and micro manipulators. Similar pieces can be bought for several hundreds of dollars but not with the degree of customization required.

I printed an adapter to hold a red filter over a lamp used for dissection. This filter is needed to prevent the bleaching of pigment in retinal tissues during tissue handling so that the responses of these cells to light can later be measured. I might have been able to find a commercially-available adapter, but this way, I was able to get exactly what I needed in a timely manner, and the only cost was my time.

We are using 3D printing to fabricate custom parts for an open-source operant chamber that can be used to measure how willing mice are to work for rewards like sucrose. While multiple commercial operant chambers exist, they cost several thousand dollars. We have developed one from open-source and off the shelf parts that costs around $100 in total... While we have been able to repurpose many household objects for our operant chamber, we turned to 3D printing for parts that were not possible to find. We are currently printing custom covers for the sensors, and a drinking well that we can combine with the photo interrupter switch to track when the mouse drinks the sucrose. All of our models will be available online for others to use. We hope that our open source operant chamber will make behavioral research more affordable for scientists across disciplines.

Most (75%) respondents were willing to pay to print 3D models after the free pilot, although we continued to provide our 3D printing service for free after it became permanent. There was high interest in 3D scanning (76%), modeling (72%), and training (62%). Half of the respondents heard about the pilot via word of mouth (50%). People reported being very satisfied with the service (average 4.75 out of 5) and very likely to recommend it to their colleagues (average 4.88 out of 5). Respondents also reported a high likelihood of printing on a monthly basis (63%).

## DISCUSSION

Even with printer breakdowns and prints that did not always come out correctly the first time, the 3D printers had a definite “coolness” factor, especially among younger researchers. The service garnered positive NIH campus media attention for the library in the form of articles in the *Catalyst* and *NIH Record* newsletters and other publications: The library’s first 3D printer customer is getting a patent for her design, and a paper was published about the mouse operant chamber [25]. Furthermore, several labs have tried out the library’s printers and purchased their own. The advice and resource sharing generously given by the partners significantly contributed to the 3D-printing project’s success. For example, a partner who had two Makerbots in his lab provided repair advice, and others printed models requiring advanced features.

Consumer-level 3D printing is a new technology that can break down and require repairs, but most repairs are not difficult. It is highly recommended that libraries choose a printer with an extensive online community, as most common problems and their solutions have been encountered before by others who have documented them on YouTube, which is an invaluable resource in this field [17]. Extended warranties and maintenance agreements are encouraged. If a library can afford one, it should consider an entry-level professional printer ($15,000–$20,000) as they are more reliable and have better service contracts.

Only a tiny minority of orientation participants knew anything about how to model or print in 3D. They had seen news stories about 3D printing and were excited about the new technology, but the modeling software learning curve is steep and requires time and motivation to master. Only an estimated 40% of orientation attendees printed models. If modeling classes had been concurrently available, more users may have followed through with print jobs. Of those who did print models, many needed advice to make their models printable. Consequently, in 2016, the library again tapped into the rich resources of the NIH community and found volunteers to teach various beginner-level 3D-modeling tools—including Blender, Solidworks, OpenSCAD, and Maya—and molecular modeling tools such as Chimera.

When the library decided to offer 3D printing on a long-term basis, a more reliable printer, a Stratasys uPrint, was purchased at the end of 2015. It is hoped that there will be less down time, and scientists have been particularly interested in the uPrint’s dissolvable supports, especially when printing delicate protein ribbon structures. The uPrint utilizes a chemical bath to soak away supports, because manually picking away supports is tedious work that can break delicate models. The uPrint also has a sealed, heated chamber that reduces curling and cracking of models, which can be issues with consumer-grade printers.

## CONCLUSIONS

3D printing is a rapidly growing area that currently touches nearly every area of medicine: custom implants, dentistry, surgical guides, prosthetics, facial reconstruction, and tissue engineering for drug testing. The demand for 3D-printing skills will continue to grow as there are new developments in microprinting and tissue engineering. Health sciences libraries can position themselves as training centers where doctors and medical students, as well as basic scientists, can learn about the scope of this technology and how to leverage it in their work to improve patient care. Those libraries lacking the staff or funding for a 3D printer could offer 3D modeling training. Many software packages are free, and the number of commercial print services where patrons can send their models is growing.
